# Compound Crises: The Impact of Emergencies and Disasters on Mental Health Services in Puerto Rico

**DOI:** 10.3390/ijerph21101273

**Published:** 2024-09-25

**Authors:** Fernando I. Rivera, Sara Belligoni, Veronica Arroyo Rodriguez, Sophia Chapdelaine, Varun Nannuri, Ashley Steen Burgos

**Affiliations:** 1 Puerto Rico Research Hub, University of Central Florida, Orlando, FL 32816, USA; sara.belligoni@ucf.edu; 2 School of Medicine, University of Connecticut, Storrs, CT 06269, USA; 3 School of Psychology, Appalachian State University, Boone, NC 28608, USA; 4 College of Medicine, University of Central Florida, Orlando, FL 32816, USA; 5 School of Behavioral and Brain Sciences, Ponce Health Sciences University, Ponce, PR 00716, USA

**Keywords:** disasters, emergencies, mental healthcare, mental health services, Puerto Rico, Puerto Ricans

## Abstract

Background: Mental health in Puerto Rico is a complex and multifaceted issue that has been shaped by the island’s unique history, culture, and political status. Recent challenges, including disasters, economic hardships, and political turmoil, have significantly affected the mental well-being of the population, coupled with the limitations in the accessibility of mental health services. Thus, Puerto Rico has fewer mental health professionals per capita than any other state or territory in the United States. Objective: This comprehensive review examines the impact of disasters on mental health and mental health services in Puerto Rico. Given the exodus of Puerto Ricans from the island, this review also provides an overview of mental health resources available on the island, as well as in the continental United States. This review identifies efforts to address mental health issues, with the intent of gaining a proper understanding of the available mental health services, key trends, as well as observable challenges and achievements within the mental health landscape of the Puerto Rican population. Design: A comprehensive search using the PRIMO database of the University of Central Florida (UCF) library database was conducted, focusing on key terms related to disasters and mental healthcare and services in Puerto Rico. The inclusion criteria encompassed studies on Puerto Rican individuals, both those who remained on the island and those who migrated post-disaster, addressing the mental health outcomes and services for adults and children. We included peer-reviewed articles published from 2005 onwards in English and/or Spanish, examining the impact of disasters on mental health, accessibility of services, and/or trauma-related consequences. Results: In this scoping review, we identified 39 studies addressing the mental health profile of Puerto Ricans, identifying significant gaps in service availability and accessibility and the impact of environmental disasters on mental health. The findings indicate a severe shortage of mental health services in Puerto Rico, exacerbated by disasters such as Hurricanes Irma and Maria, the earthquakes of late 2019 and early 2020 that followed, and the COVID-19 pandemic, resulting in substantial delays in accessing care, and limited insurance coverage, particularly in rural regions. Despite these challenges, efforts to improve mental health services have included substantial federal funding and community initiative aimed at enhancing care availability and infrastructure. Limitations include the use of a single database, language restrictions, and potential variability in data extraction and synthesis. Conclusions: This scoping review highlights the significant impact of disasters on mental health in Puerto Rico and the challenges in accessing mental health services exacerbated by disasters. Despite efforts, significant gaps in mental healthcare and services persist, emphasizing the need for more rigorous research and improvements in infrastructure and workforce to enhance mental health outcomes for Puerto Ricans both on the island and in the continental United States.

## 1. Introduction

The existing body of literature concerning the health profile of Puerto Ricans has consistently identified the lack of availability and accessibility of health services for both physical and mental health [1]. The landscape of mental health within Puerto Rico can best be illustrated by a 2016 report from the Administration of Mental Health and Anti-Addiction Services (ASSMCA), which found that 7.3% (165,497) of Puerto Ricans between the age of 18–64 suffer from a serious mental illness. The most prevalent mental illnesses were anxiety and mood disorders. Women are reported to be more likely to experience serious mental illness (4.2%) compared to men (3.1%); similarly, women are more likely to be affected by a psychiatric disorder (10.5%) compared to men (8.5%). Overall, statistical significance was also found when the authors of the report tested for other disorders: women are more likely to suffer from generalized anxiety disorder, major depressive disorder, and dysthymia compared to men. Conversely, men are more likely to experience severe forms of attention deficit hyperactivity disorder (ADHD) compared to women, with almost 1% of the male Puerto Rican population being affected by ADHD. Moreover, men show higher rates of substance use disorder, with 2.1% versus 1.2% for women when it comes to any drug use disorder. Men are more likely to be affected by alcohol use disorder, with 4.2% versus 1.5% for women; similarly, men record 3.4% for nicotine disorder compared to 1.8% in women. The elderly population was also at a greater risk for having a mental health illness, since approximately 19.7% of this population suffered from depression [2].

The mental health profile and lack of services were exacerbated after the impact of Hurricanes Maria and Irma in September of 2017. Past literature suggests that direct physical impact and the loss of resources in the recovery environment characterize the disasters’ impacts on mental health [3]. After a disaster, the affected population may experience post-traumatic stress disorder (PTSD), with gradually dissipating symptoms [3]. A small percentage of the affected population developed psychopathologies and often suffered from long-lasting symptoms that impaired every sector of their livelihoods, greatly diminishing their work, love, and individual quality of life. According to the Mayo Clinic, some long-lasting symptoms may include, as shown in the case of Puerto Ricans who experienced a multitude of complex emergencies, trouble sleeping, eating disorders, and anxiety [4,5]. In the case of Puerto Rico, the mental health services were unable to keep up with the demands of those dealing with the mental health consequences of the disaster [4].

### 1.1. Methodological Approach

Given these multifaceted issues, we examined the existing literature on the mental health profile of Puerto Ricans to identify gaps in the availability and accessibility of services and understand the broader impacts of environmental disasters on mental health. The articles for this paper were identified through a comprehensive search using the Primo Database in 2023 and 2024. The search strategy included a combination of key terms relevant to this study’s focus. Specifically, the following terms were used: “mental healthcare”, “mental health services”, “disasters”, “Puerto Rico”, “Puerto Ricans”, and “trauma”. Ideas were also drawn from the study *Mental Health of Puerto Ricans Who Stayed in Puerto Rico Compared to Those Who Migrated to Florida after Hurricane Maria* [6].

The inclusion criteria were designed to capture a comprehensive and relevant set of studies. We included studies focusing on Puerto Rican individuals, both those who remained in Puerto Rico and those who migrated elsewhere, such as Florida, following disasters such as hurricanes. The studies needed to address mental health outcomes and/or services for both adults (18+) and children. We also included research examining mental healthcare and/or services, including their accessibility and/or availability, and studies exploring the impact of disasters, such as Hurricane Maria and Hurricane Irma, on mental health. Relevant studies were screened based on titles and abstracts, followed by full-text reviews to confirm eligibility. Through this systematic approach, this study aims to provide a comprehensive understanding of the mental healthcare landscape for Puerto Ricans, highlighting the impact of disasters and identifying areas for improvement.

Five reviewers independently screened the sources of evidence identified through the search strategy. Each reviewer assessed the titles and abstracts of the identified records for relevance based on the predefined inclusion and exclusion criteria. Discrepancies in the initial screening were resolved through discussion and consensus. Following this, the full texts of potentially relevant studies were retrieved and evaluated for eligibility by the same reviewers. Each reviewer independently assessed the full texts to ensure that they met the inclusion criteria, focusing on studies addressing mental healthcare and services for Puerto Ricans, the impact of disasters, and trauma-related mental health outcomes. Any disagreements during the full text review were resolved through discussion among all reviewers, with a final decision made by majority vote if consensus could not be reached. After consensus was reached, a total of (*n* = 39) papers were included in the examination of the literature used in this paper.

### 1.2. Results

This paper first examines the literature on mental healthcare and services before and after Hurricanes Maria and Irma. Those trends are then contrasted with mental health services offered both in Puerto Rico and the continental United States. We then observe these patterns within the context of the consequences inflicted by the COVID-19 pandemic. Lastly, we identify knowledge gaps in the literature and summarize the status of mental healthcare and services in Puerto Rico.

## 2. Mental Healthcare and Services before Hurricane Maria in Puerto Rico

Even before Hurricane Maria hit Puerto Rico in 2017, Puerto Ricans were at a greater risk for poor mental health outcomes [2]. In a study, Chapdelaine (2022) examined mental health trends in Puerto Rico via a mixed method analysis employing content analysis of newspaper articles and interviews. The investigation found that Puerto Ricans are more likely to develop inadequate mental outcomes. A 2016 report by ASSMCA found that anxiety and mood disorders were the most frequent disorders seen in Puerto Ricans even prior to Hurricane Maria [2,6].

A group of scholars conducted a study in Puerto Rico to assess the availability of mental health crisis services offered in Puerto Rico before Hurricane Maria. They looked at psychiatric emergency walk-in clinics, suicide prevention services, and crisis intervention teams’ availability as of 2016. The number of facilities providing services for psychiatric emergency walk-ins was 34, and the percentage was 38.6%. As for the number of facilities providing services for suicide prevention, this came to 66, and the percentage was 75%. In addition, the number of facilities providing services for crisis intervention teams was 60, and the percentage was 68.2%. They also assessed the availability of mental health crisis services offered to adults who do not have healthcare insurance [7]. The provision of psychiatric emergency walk-in services for uninsured patients was observed in 22 facilities, representing 25.6% of the total number of facilities. A total of 38 facilities were identified as providing suicide prevention services to uninsured patients, encompassing 44.2% of the total. Finally, services related to crisis intervention teams for uninsured individuals were available in 30 facilities, constituting 34.9% of the overall total [8,9] (For more information about crisis intervention and suicide prevention centers in Puerto Rico, visit the 988 Suicide & Crisis Lifeline at https://988lifeline.org/crisis-centers-by-state-and-u-s-territory/ (accessed on 15 September 2024) or Linea PAS at https://lineapas.assmca.pr.gov/ (accessed on 15 September 2024).

Based on these data, it is evident that access to mental health crisis services was deficient prior to Hurricane Maria, particularly for individuals lacking healthcare insurance. Medicare/Medicaid coverage, the island’s public healthcare system, and Puerto Rico’s economic profile were additional factors, besides the lack of accessible services, that further impacted the access to mental healthcare for Puerto Ricans. A change in the healthcare system occurred with “La Reforma”. The system reform was put in place in 1993 under the direction of then-Governor Pedro Juan Rosselló González. This shift resulted in public hospitals and clinics being taken over by private organizations. Furthermore, doctors were told to collaborate part-time with Medicare and Medicaid healthcare organizations and prioritize treating individuals with insurance [10]. Another public healthcare system that Puerto Ricans utilize is “Mi Salud”, which is mainly reimbursed through Medicare and Medicaid. Studies indicate that 94% of Puerto Ricans are insured and “of this proportion, only 30% receive health insurance privately via an employer, and the rest are completely reliant on the underfunded Mi Salud system”. Although the healthcare coverage was adequate before Hurricane Maria, poor health was evident. Many individuals had disabilities, diabetes, and HIV and were not properly treated, which resulted in high death rates [10].

A significant determinant in accessing mental health services in Puerto Rico was the financial insufficiency supported by health insurance companies, specifically Medicare/Medicaid. Unlike the continental United States, the Medicare/Medicaid fund available to patients in Puerto Rico has a debt ceiling. Once that ceiling is reached, coverage for healthcare services can no longer be guaranteed. As a result, providers are not paid for services, and patients are left with a financial burden [11]. Moreover, funding for mental healthcare can be subject to changes and adjustments through legislative actions and budget allocations by the U.S. Congress, where Puerto Rico has limited representation and no voting rights [12].

## 3. Mental Healthcare and Services after Hurricane Maria in Puerto Rico

Hurricane Maria shed light on the pre-existing mental health crisis in Puerto Rico. The aftermath of the hurricane greatly impacted the mental health of those who suffered direct impacts, with children, students, and first responders being at the greatest risk. Addressing the immediate distress after a disaster can prevent mental health illness from developing. Unfortunately, modes of prevention were not implemented and were not readily available in the aftermath of Maria. The severity of distress present in Puerto Rico resulted in an increase in the suicide rate, with the suicide rate post-Maria increasing by 55% between September and December 2017 [13,14]. The Puerto Rican suicide hotline, Linea PAS, received double the number of calls in the 6 months following Hurricane Maria [15].

The trauma of the hurricane and its aftermath also had long-term effects on the mental health of children and youth in Puerto Rico. Out of the entire population, children were at the greatest risk of developing mental health psychopathologies. Many children and youth experienced symptoms of PTSD, depression, and anxiety in the months following the hurricane, and these symptoms persisted over time [16]. In the aftermath of Hurricane Maria, the children attending public schools were given a survey that explored the stressors due to hurricane-related PTSD and depression. The results indicated that 6.6% experienced the death of a loved one, 29.9% had suicidal thoughts, 45.7% experienced damage to their home, 31% experienced damage to their belongings, 25.5% were required to relocate, 83.9% witnessed house damages, 5.7% switched schools, and 57.8% had friends or family that relocated. In addition, 7.2% of youths would have met the criteria for a PTSD diagnosis after Hurricane Maria [17].

As of 30 September 2018, approximately one year after Hurricane Maria, the Health Resources and Service Administration (HRSA) recorded that 72 out of 78 municipalities in Puerto Rico were ‘medically underserved areas’. Despite these challenges, there have been efforts to address mental health issues for Puerto Ricans. Acknowledging the lack of available resources, several community outreach groups, as well as institutional partnerships, were formed. Three key resources are the HRSA, Better Help App, and Centros de Apoyo Mutuo (CAM). In Puerto Rico, HRSA provides healthcare services to individuals who are the most at risk and unable to afford quality services. These individuals include people with HIV/AIDS, new mothers, and low-income families. HRSA also assists with healthcare services in rural settings and other areas where help is lacking [18]. Alternatively, the Better Help app is available globally and matches users to an online counselor based on their needs, such as stress and anxiety. This app is convenient because it takes away the stigma from in-person therapy, it is easily accessible through desktop or smartphones, and it is more affordable than traditional therapy [19]. Centros de Apoyo Mutuo (CAMs) are grassroots, self-managed organizations that have sprouted throughout Puerto Rico following FEMA’s insufficient and lackluster response to Hurricane Maria [20]. These centers are focused solely on helping the communities they are working in, straying away from hierarchy and competition. The first CAM was created in Caguas, where it provided breakfast and lunch three times a week to volunteers who came to help with other activities. Among the latter, CAMs throughout the island engaged in debris-cleaning operations, distribution of food and water, installation of blue tarps, and more [21].

According to an article, “People sought consultation for a mental health problem from a general medical provider (31.5%), family member and friends (23.6%), followed by a mental health specialist (17.5%) or clergy”. Many individuals with mental health issues in Puerto Rico turn to primary care services and non-psychiatric hospitals for treatment. Most people also went to the emergency room for mental health issues because general hospitals could also provide extensive healthcare services, which several people needed. Due to the lack of mental health services available, the Clinical Psychology Services Program (CPSP) was created. Their key mission was to provide inclusive services for all individuals, focusing on their specific physical and mental health needs. Their services include medical and outpatient referrals for mental healthcare services, screenings, and follow-up visits for those who require it [20,22].

Other support systems for Puerto Ricans include schools and families. Several universities in Puerto Rico supported the resilience of their students, including the Inter American University of Puerto Rico-Arecibo, Universidad Ana G. Mendez-Gurabo, University of Puerto Rico-Mayaguez, University of Puerto Rico-Rio Piedras, and University of Puerto Rico-Utuado. These universities provided access to meals through local food pantries and financial assistance during the COVID-19 pandemic. They also provided students with in-person and virtual counseling services. In addition, they provided resources for post-graduation, including internship and research opportunities and job fairs [21]. As for family support, many Puerto Rican families supported each other through similar spiritual beliefs and practices. These practices allowed families to create stronger bonds with each other and an increase in faith. The key factor was that families came together to create a positive outlook by remaining hopeful about the future [23].

## 4. Mental Healthcare and Services in the Continental United States

The devastation caused by Hurricane Maria led many to move out of the island to the continental United States. In many cases, there were higher rates of reported PTSD among Puerto Ricans who moved to Florida because they lost almost everything on the island, and they had to adapt to a new life in the 50 states and the District of Columbia. However, both Puerto Ricans who moved and those who stayed on the island experienced trauma and anxiety, with the rates of PTSD being 65.7% in Florida and 43.6% in Puerto Rico [24].

The Puerto Rican culture can be described as hopeful, resilient, and overall altruistic. This central thread is in direct opposition to the individualistic collective that can be observed in the 50 states and the District of Columbia. This cultural difference made it harder for individuals who moved after Hurricane Maria to cope with mental stressors. In a studyfound that 65% of non-metropolitan counties in the United States do not have a psychiatrist, and 60% of the rural population live in areas with a shortage in mental health providers [23,25]. Those who felt a sense of abandonment found themselves trapped in a constant cycle of thinking that they can take care of themselves, even though the stigma surrounding mental health decreased.

The Hispanic health paradox states that although Hispanics are at a greater risk for poor physical and mental health, they tend to have a greater life expectancy compared to other groups [7]. However, this phenomenon does not apply to Puerto Ricans, specifically those that reside in the 50 states and the District of Columbia [21]. Puerto Ricans do not benefit when moving or living in the continental United States, since they no longer have the same support system they usually have while living on the island [26]. This same population also suffers from disadvantageous socio-economic status conditions [5].

A recent study led by the Urban Institute and the University of Central Florida (UCF) analyzed the receiving community capacity of Central Florida after Hurricane Maria, to further understand if the region was ready to receive such a large influx of people. The data gathered during the study involved interviewing providers from Central Florida. These interviews provided several insights, and as one of the interviewees mentioned about the experience of Puerto Ricans moving to Florida, “*You have no job, you might not have any family, you don’t speak the dominant language. So, all those things I think are compound, and what we know about stressors in general is that they can increase symptoms in people who already have mental health disorders and physical disorders. Or they may create disorders in people who didn’t have disorders in the first place, so we worked with several people who were depressed because they lost their home in Puerto Rico*”. *Another* interviewee explained, “*Healthcare for people without insurance is always a tough one [...] there are great free clinics, awesome opportunities for people to get help, but obviously there’s a waiting list, eligibility requirements, proof of income...hoops that are put in place, for good reason, for eligibility, mostly related to funding sources*”. Another interviewee mentioned that federally qualified health centers are a good resource. “*In my experience, the only sliding care providers are federally qualified health centers, which are non-profit, and they tend to have a pretty rigorous standard but at the same token, they also tend to have a long waiting list because they are federally qualified health centers and will see the uninsured with little to no money*”. In addition, another interviewee stated, “*One thing that I saw provided was mental health counseling because I was able to go to a couple trips and I also saw what this congressional office and what Vamos4PR was also doing and they were trying to find entities out there that can help people with mental counseling as a component of possibly all other services that people need*” [6,22].

## 5. Compound Crises: Hurricane Maria, the COVID-19 Pandemic, and Their Impact on Mental Healthcare and Services in Puerto Rico

In Puerto Rico, the impact of Hurricane Maria on mental health was exacerbated by existing challenges in the mental healthcare system, including a shortage of mental health professionals and limited access to care, particularly in rural and underserved areas. March 2020 was when the first case of COVID-19 appeared in Puerto Rico. This resulted in a lockdown across the whole island, individuals losing their jobs, and schools closing. Children were at an increased risk of trauma exposure due to ongoing anxiety from Hurricane Maria. Individuals exposed to traumatic events are at a greater risk for becoming depressed, developing behavioral issues and suicidal thoughts, and coping through substances. Exposure to traumatic events may also lead to students doing poorly in school and overall poor health [17].

The COVID-19 pandemic put Puerto Ricans under a new set of stressors while they were still recovering from two prior disasters. The pandemic affected an already fragile mental health landscape due to Hurricane Maria and the 2019–2020 earthquakes. Studies have shown that the COVID-19 pandemic increased the population’s feelings of anxiety, depression, restlessness, abandonment, isolation, suicidal ideations, and abuse. These are the same psychopathologies that were observed after Hurricane Maria and during the 2019–2020 earthquakes.

As highlighted in the literature, minority populations may experience mental health disorders because of what has been defined as ‘acculturative stress’. Indeed, in addition to challenges faced on an everyday basis, those who also experienced migration can go through multiple stressors due to the need of adapting to a new culture, which may further trigger some mental health disorders. Studies found that social support can decrease the likelihood of experiencing acculturative stress, which is why several Puerto Ricans relocating after Hurricane Maria moved to areas where they had family members or friends [27]. Many Puerto Ricans who migrated to Florida after Hurricane Maria struggle with the language barrier. Due to schools closing after COVID-19, some parents were not able to assist their children with schoolwork because they did not understand English. A group of researcher conducted a study investigating this issue and interviewed people to gain insights into the challenges many faced when the pandemic hit, which were also a representation of their overall socio-economic struggles. A 27-year-old stay-at-home mother of four children shared her struggles. She explained that she can only understand English if you speak slowly and use basic vocabulary. In addition, her children were in a program at school called English as a Second Language (ESL), which helped them learn English. She said staying at home affected their reading and grammar because they were unable to have one-on-one time with their teacher who would take them aside if they needed extra help. Misty, who is a 31-year-old mother, described a similar relationship with her daughters. She explained that home schooling had affected her relationship with her daughters because the language barrier made her feel lost. Furthermore, she felt as if she was going backwards and had to re-learn certain concepts. Lydia, a 36-year-old mother and health worker, described the difficulties her family faced after the pandemic. She explained that her family ended up developing feelings of depression and anxiety, especially her two children, who had to stay home unsupervised while she worked. The transition to online classes was also difficult for her children, and they had trouble sleeping at night due to stress. Her thirteen-year-old son struggled the most with the concept of staying at home because he was used to going out with his friends after school. Both of her children gained weight due to stress eating and not having Lydia supervise their eating habits while she was at work [28].

Contrary to the observations made after Hurricane Maria, reports indicate that individuals living in Puerto Rico experienced greater difficulties than those living in the 50 states and District of Columbia. Social support is a great way to help cope with mental illness. The pandemic made this difficult, as it resulted in supportive services for mental health illnesses shutting down, leading to over reliance on support from family members, who were sometimes distant because of the pandemic restrictions on gatherings, except for the closest family members living in the same household. Newly appointed caregivers began experiencing physical and emotional overload. Furthermore, the individuals who suffered from a mental illness reported an increase in behavioral changes due to an alteration in their daily routine. These behavioral changes were further exploited by the fear associated with the pandemic. Puerto Rico reported an increase in feelings of fear, desperation, and guilt [25].

There was no reprieve for Puerto Ricans, specifically those residing on the island, as they experienced successive disasters. Hurricane Maria in 2017, the 2019–2020 earthquakes, the COVID-19 pandemic, and Hurricane Fiona in 2022 exposed a mental health crisis that is far from being resolved. These events resulted in traumatized island residents, leading to the decimation of the infrastructure and families being torn apart. It was estimated that 46% of the 100,000 young Puerto Ricans surveyed after Hurricane Maria declared that they had some sort of damage to their homes, 32% had issues with accessing food or water, 58% had a loved one leave the island because of the disaster, and 30% felt their lives were at risk. ASSMCA received nearly 3000 calls in the five days after Hurricane Fiona made landfall, as it also happened to fall on the five-year anniversary of Hurricane Maria [17].

## 6. Discussion

In this scoping review, we identified 39 studies addressing the mental health profile of Puerto Ricans, identifying gaps in the availability and accessibility of services, and understanding the broader impacts of environmental disasters on mental health. Our findings indicate that the mental health landscape both in Puerto Rico and in the 50 states and the District of Columbia was lacking in available and accessible resources and remains so today. In Puerto Rico, each disaster, starting with Hurricane Irma, has only continued to exploit an already fragile mental healthcare industry. This, along with the social stigma associated with mental health diseases, makes finding and receiving treatment a challenging task. The shortage of mental health services became worse after Hurricane Maria and magnified pre-existing struggles. Some of these concerns include a lack of accessible resources, an absence of affordable care, suicidal ideations, depression, and anxiety. COVID-19 had a significant impact on Puerto Ricans, especially on young adults.

There is a clear lack of mental health providers and services in Puerto Rico, and few of those services available are covered by medical insurance. This lack of availability and accessibility has aggravated the condition of many individuals who suffer from psychopathologies and has further devastated the mental health landscape. In 2022, the Administration de Seguros de Salud in Puerto Rico reported 15 municipalities without psychologists and 32 municipalities without psychiatrists, with the most affected municipalities being concentrated in the central and northeastern regions of the island. In a recent interview with El Nuevo Dia, Karen Martinez, the Director of Psychiatry Program at University of Puerto Rico-Medical Sciences Campus, highlighted the patients’ struggle to receive mental health services. Many patients are waiting 6–8 months to receive an appointment, and those appointments are mostly conducted over telehealth services. Receiving treatment is another challenge, as the follow-up and maintenance plan curated for patients with mental health illnesses is inconsistent at best.

Although the situation is dire, strides have been made in the right direction. Puerto Ricans mainly access healthcare through insurance, such as Medicaid and Medicare, and nonprofit organizations. As mentioned previously, community outreach groups and institutional partnerships have been made to bridge the gap present in the mental health landscape. In May 2023, the Federal Emergency and Management Agency (FEMA) awarded Puerto Rico nearly 80 million dollars to aid in the recovery of mental health facilities, specifically those supplied by ASSMCA. About USD 31 million of the budget has been used to repair about 20 facilities, ranging from recovery facilities in Moca, the Drug Courts in Arecibo, to the Mental Health Center in Mayagüez [29]. Furthermore, problems related to lead and asbestos exposure have been solved. In addition, the Financial Oversight and Management Board for Puerto Rico had a meeting on 30 June 2023 to discuss the consolidated budget for the 2024 fiscal year. The overall budget that Governor Pedro R. Pierluisi signed on 29 June 2023 amounts to USD 12,747,000 and went into effect on 1 July 2023, with the following categories: USD 307,000 toward families and children, USD 876,000 toward economic development, USD 980,000 toward pension reserve trust, USD 1,102,000 toward debit service, USD 1,379,000 toward health, USD 1,381,000 toward public safety, USD 2,100,000 toward retiree payments, USD 2,195,000 toward education, and USD 2,427,000 toward other areas. This general fund budget, along with the USD 5 million special revenue fund budget and the USD 13.3 billion federal fund budget, was approved by the Oversight Board, which means that USD 31 billion is the total consolidated budget for the Puerto Rico government [30]. Through grant funding, in 2023, the Substance Abuse and Mental Health Services Administration (SAMHSA) allocated USD 20,463,667 toward mental health services and USD 12,672,025 toward substance abuse services, for a total of USD 33,135,692. This money is then divided among networks within the municipalities, such as non-profits and hospital systems, to help advocate for prevention, treatment, and education. For example, Rehaciendo Comunidades con Esperanza in Rio Piedras has been allocated USD 234,986 of the funds to educate 3500 Spanish-speaking Puerto Ricans over five years on the detection and prevention of mental health issues and to increase the overall awareness of the resources available [31].

The following study limitations should be acknowledged to better contextualize this scoping review and highlight areas for further research and potential improvements in future studies. The search was conducted using only the UCF library database, which might not capture all relevant studies. Important studies indexed in other databases could have been missed. Furthermore, this review includes studies published from 2005 onward. While this ensures relevance to current practices, it may exclude older studies, which could provide a valuable historical context. The review focused on the Puerto Rican population, both on the island and those who migrated. While this specificity provides in-depth insights into this group, the findings may not be generalizable to other populations. Finally, limitations in data extraction and data synthesis in this scoping review should be noted, as multiple reviewers worked independently to screen studies and provide a descriptive synthesis of these studies. Differences in interpretation could have introduced variability in the data extraction and synthesis.

## 7. Conclusions for Future Directions

This scoping review provides a comprehensive overview of the mental health landscape in Puerto Rico, highlighting the significant impact of disasters on mental health and the challenges in accessing mental health services. The findings underscore the complexity of mental health issues on the island, exacerbated by economic hardships, political instability, and natural disasters such as both Hurricanes Maria and Irma and the COVID-19 pandemic. This review also sheds light on the mental health resources available to Puerto Ricans who have migrated to the continental United States. Despite the efforts to address these issues, there remain significant gaps in mental healthcare and services, particularly concerning accessibility and availability. The heterogeneity in study designs and the varying quality of the included studies emphasize the need for more rigorous research to develop a clearer understanding and effective interventions. Future research should focus on longitudinal studies and include a broader range of sources to capture the full spectrum of mental health challenges and resources for the Puerto Rican population.

Moving forward, it is evident that more research needs to be conducted to fully understand the extent of available and accessible mental health services both in Puerto Rico and the continental United States. There are a few overall industry recommendations. One is to strengthen the workforce, such as offering initiatives to alleviate the mass exodus of physicians. Another is to place a bigger focus on the island’s overall infrastructure, starting with a better transportation system; the struggle with the island’s transportation system contributes to mental health psychopathologies, such as anxiety and distress. In addition, there is a lack of publicly available data, and more studies should be conducted in the future to gain more insight. As for the literature review, there is a lack of information on mental healthcare and services before Hurricane Maria, and it is clear that meaningful change will not take place unless action is taken.

## Data Availability

Not applicable.
